# The Mechanical Properties of Breast Cancer Cells and Their Surrounding Microenvironment

**DOI:** 10.3390/ijms26115183

**Published:** 2025-05-28

**Authors:** Leila Jahangiri

**Affiliations:** 1 School of Science and Technology, Nottingham Trent University, Clifton Site, Nottingham NG11 8NS, UK; leila.jahangiri@ntu.ac.uk; 2 Division of Cellular and Molecular Pathology, Department of Pathology, Addenbrookes Hospital, University of Cambridge, Cambridge CB0 2QQ, UK

**Keywords:** breast cancer, mechanical forces, extracellular matrix, invasion

## Abstract

Breast cancer is a major health concern for women worldwide, and therefore, understanding various changes acquired by breast cancer cells is relevant to a better comprehension of the disease. One such change includes alterations to the mechanical properties of breast cancer cells. For example, cells with high malignant potential show lower adhesion forces and higher cell deformability. Mechanical forces, including tensile and compressive forces of the cytoskeleton and the extracellular matrix such as integrin, collagen, and the basement membrane, can affect BC cells. These forces alter the properties of cancer cells, drive them towards invasiveness due to different motility and proliferative profiles, and change their microenvironment. This study will focus on the mechanical characteristics of breast cancer cells and the extracellular matrix. Furthermore, changes induced in breast cancer cells following exposure to mechanical forces will be reviewed. Genes that link phenotype to mechanical forces and the implications of these forces for diagnostics and treatment will be discussed.

## 1. Breast Cancer Cells and Their Mechanical Properties

Breast cancer (BC) is a major health problem for women, and close to 13% of this group will be diagnosed with BC in their lifetime, while BC contributes to circa 25% of all cancers and 16% of cancer-related deaths in this group in the western world [1,2]. BC can be categorised based on immunohistochemical profiles and hormone receptor expression as oestrogen receptor-positive (ER+), progesterone receptor-positive (PR+), triple negative (TN−), and human epidermal growth factor receptor-positive (HER2+). On these grounds, 4 distinct subtypes have been described, including luminal A and B, TN−, and HER2+ [3]. Accordingly, luminal A are usually ER+ and/or PR+, HER2−, and luminal B are usually ER+ and/or PR+, HER2+/−. Further, the HER2+ category is ER−, PR−, and HER2+, and the TNBC subtype is negative for all three hormone receptors [4]. BC can be categorised into stages, akin to other cancers, which include in situ (0), signifying non-invasive BC. This is followed by early-stage invasion (IA, IB, IIA, and IIB) when limited invasion and lymph node involvement have been established. In addition, later stages are locally advanced (IIIA, IIIB, and IIIC), in which wider spreading in the chest and metastasis has occurred (IV) [5]. Furthermore, solid tumours such as BC will display altered mechanical properties compared to their normal tissue counterparts. These mechanical properties include stiffness, rigidity, adhesion, traction, and deformability (elasticity). In addition, as the tumour progresses from its in situ state and carries out invasion and metastasis, the mechanical properties of BC cells can become more complex [6]. This background exemplifies the need to understand the mechanical deviations from the homeostasis state of the breast tissue during malignant transformation.

**Figure 1:**
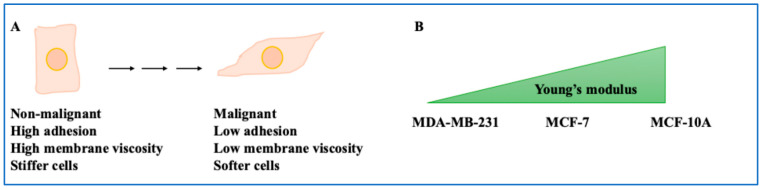
The mechanical properties of BC cells. (**A**) Non-malignant cells have low cell elasticity (stiff cells) but high membrane viscosity and adhesion. In contrast, malignant cells have high elasticity (soft cells) but low membrane viscosity and adhesion. (**B**) The non-malignant cell line MCF-10A has the highest Young’s modulus, followed by MCF-7 and MDA-MB-231.

A good place to start is the mechanical properties of breast cells and their malignant counterparts. One aspect that impacts these mechanical properties is gap junctions. Gap junctions comprise connexin proteins and are major routes of intracellular communication between cells. These gap junctions allow the exchange of intracellular signalling molecules such as cAMP and metabolites and ions such as Ca^2+^ between cells [7]. A study aimed to understand the mechanical properties of BC cells and their tight junctions [8]. For example, the role of gap junction connexin 43 (Cx43) in adhesion forces was assessed by fluidic force microscopy for single cells or cell clusters. Cx43 expression in MDA-MB-231 single cells did not affect adhesion forces (70 Nanonewton, nN). In Cx43-expressing cells grown in clusters, forces increased (380 nN). This value was 145 and 300 nN for Cx43 knockdown MDA-MB-231 and wild-type cells grown under the same conditions, respectively. This outlined the role of Cx43 and population density in increasing cell–cell adhesion in cell clusters [8]. It showed that tight junctions can impact the mechanical properties of BC cells, such as adhesion forces. In addition, the viscoelastic properties of the cell membrane (liquidness or solidness of a substance) and the extracellular matrix (ECM) were represented by frequency shifts [8]. Rigidity and viscoelasticity of the cell membrane of detaching cells were linked to dissipation shifts in quartz crystal microbalance methods. Non-invasive cells were stiff and had viscous membranes and stiffer cytoskeletons, while metastatic cells were softer and had lower viscous membranes and soft cytoskeletons [8,9]. The higher the malignant potential, the lower the adhesion and the greater the cell deformability (Figure 1A).

Another study confirmed this using single-cell force spectroscopy and quartz crystal microbalance with dissipation. MCF-7 showed greater adhesion, intracellular forces, and stiff phenotypes than their MDA-MB-231 counterparts [10]. Another study exposed MCF-10A, MCF-7, and MDA-MB-231 to 1 nN of force. The non-malignant cell line MCF-10A had the highest Young’s modulus (representing stiffness), followed by MCF-7 and MDA-MB-231 [11]. As for viscosity, MCF-10A demonstrated the highest viscosity, while MCF-7 and MDA-MB-231 showed lower values. MCF-10A cells displayed an organised cytoskeleton (with actin aligned as stress fibres), while MCF-7 and MDA-MB-231 cells had more amorphous actin (Figure 1B) [11]. Finally, the ZR-75 cell line, which is hormone-dependent, and BT-20, which is TNBC, were investigated using atomic force microscopy and optical nanomotion. Moreover, 200–300 nm under the cell membrane, Young’s modulus of BT-20 exceeded that of ZR-75, while in deeper regions, this was the opposite, suggesting stiffness at indentation depths is an important factor to investigate [12,13,14]. Overall, the mechanical properties of BC cells are relevant to understanding this cancer. Table 1 summarises the aspects encapsulated in this study.

## 2. The Mechanical Properties of the ECM and the Cytoskeleton

### 2.1. Integrin and Collagen

**Figure 2:**
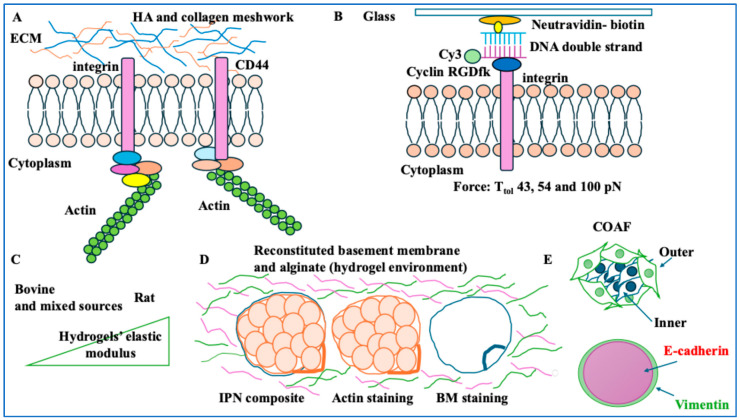
The properties of the ECM and cytoskeleton. (**A**) CD44 and β1-integrin were instrumental to the traction forces of an HA-based tumour microenvironment. (**B**) Contraption for integrin tension characterisation (tension gauge tether, TGT). A cyclin RGDfK-integrin and a Cy3 fluorescent marker dye were attached to single-stranded DNA. This single strand was complementary to another single-stranded DNA attached to a glass surface via a biotin-neutravidin complex. T_tol_, or the rupture force representing each specific TGT, was a factor of the position of the biotin tether. (**C**) Rat-based collagen hydrogels had a higher elastic modulus than bovine and mixed-source collagen hydrogels. (**D**) Organoid MCF-10A acini were harvested and encapsulated in a reconstituted basement membrane and alginate (IPNs). The composite shows an acinus with the basement membrane (BM, in blue) revealing cell invasion and basement membrane breaching (versus the orange actin staining). Upon breaching the basement membrane and the outward movement of cells, the basement membrane moved inward to the acini. (**E**) The COAF group had higher E-cadherin in the inner region and higher vimentin in their outer region; this suggested that epithelial cells were segregated in the inner region, while fibroblasts were segregated to the outer region.

The scientific community has witnessed a substantial change in perceptions of the ECM. These perceptions have evolved from viewing this matrix as an inert mesh of proteins, fibres, and proteoglycans to a highly dynamic and sophisticated 3-dimensional network of macromolecules and fibres with roles in signalling propagation, integration, and relay [35]. Given this background, we can postulate that the ECM possesses mechanical properties that affect cell growth and migration. The ECM glycosaminoglycan (GAG) and collagen 1 withstand compression and sustain tension, respectively [15]. Collagen I is an important structural protein involved in signalling and adhesion that can impact the mechanical interactions between the cell and the ECM and relies on integrin [15]. Also, integrins are heterodimeric plasma membrane receptors, which are involved in the sensing and integration of mechanical cues [36]. A study investigated how cell traction force was linked to β1-integrin and CD44 (a receptor associated with hyaluronic acid, HA, a type of GAG). Traction force is defined as a mediator of ECM-cell interactions. The authors developed 3 ECMs, including collagen I (1.5 mg/mL); collagen I (1.5 mg/mL) + HA (1.5 mg/mL), or H1; and collagen I (1.5 mg/mL) + HA (3 mg/mL), or H2. HA in H1 and H2 ECMs decreased the pore sizes and fibre diameters compared to collagen-only gels [15]. They embedded MDA-MB-231 cell lines in the ECMs and exposed the cells to fluorescently labelled beads bound to collagen (followed by cytochalasin D exposure to depolymerise actin). HA reduced traction force, and the cell body became softer than in collagen-only conditions [15]. In the MDA-MB-231 cell model, CD44 and F-actin colocalised significantly with H2 compared to H1 and collagen-only conditions. The hypothesis was that in the presence of HA, β1-integrin and CD44 were required for force generation. In the collagen-only condition, blocking β1-integrin (irrespective of CD44 status) reduced traction. Blocking CD44 or β1-integrin using antibodies in H2 conditions significantly reduced traction forces. Therefore, HA reduced traction force by modifying the architecture of the ECM and depended on β1-integrin and CD44 (Figure 2A) [15].

Integrin, as alluded to in the previous example, is a transmembrane receptor involved in cell adhesion [36]. A study investigated integrin tension under constraints and how this regulated cell migration and adhesion. To that end, they designed a tension gauge tethering (TGT) contraption consisting of microchannels attached to cyclic RGDfK as a ligand of integrin and Cy3 dye fused to a single-stranded DNA. This single strand was complementary to a piece of DNA immobilised to a surface by biotin and neutravidin bonds. The rupture force (T_tol_) was required for mechanical melting. This value was related to the position of the biotin tether and the lower DNA strand [16] (Figure 2B). Changes in Cy3 levels during adhesion and migration allowed for integrin tension detection. Calculations showed that the tethered single-stranded DNA (via the indicated biotin and neutravidin bond) would exhibit 100 piconewton (pN). MDA-MB-231 cells were grown in 43, 54, and 100 pN T_tol_ [16]. For T_tol_ of 43 pN, cells adhered but did not spread due to relatively easy loss of adhesion. For T_tol_ 54 pN, the cells spread more and became polarised by forming focal adhesion complexes (also, TGT rupture occurred along cell edges). Therefore, integrin tensions above 40 pN allowed focal adhesion complex formation in BC cells, while integrin tension exerted on focal adhesion complex at edges was within the 54–100 pN range [16]. In the presence of EGF, cells became more polarised. Further, EGF impacted integrin-modulated adhesion and cell migration. In summary, the interplay between integrin tension and the ECM affected BC cell migration [16].

Collagen-based matrixes and scaffolds are used in cancer research since collagen I is abundant in the ECM and can realistically simulate the tissue. As such, collagen hydrogels are commonly used materials for emulating the ECM. A study focused on the effect of the concentrations and characteristics of collagen on these hydrogels [17]. They used scanning electron microscopy and rheology to generate an atlas of these properties. Altering polymerisation conditions could generate varied ECMs with different fibrillar structures. Nine hydrogels of bovine, rat, and mixed origin with 0.8, 1.5, and 2.5 mg/mL of collagen were selected. High and low collagen concentrations affected the elastic modulus of the hydrogels (a measure of resistance to deformity). Rat-based collagen hydrogels gave a higher elastic modulus than their bovine and mixed-source collagen hydrogel counterparts. For instance, 2.3 mg/mL of rat-based collagen gave an elastic modulus of circa 240 Pascals, 25 times greater than that measured for 0.8 mg/mL of bovine-sourced collagen hydrogels. They grew MDA-MB-231 cells in the described hydrogels [17]. The mechanical properties of the ECM did not affect cell morphology, and BC cells adopted a mesenchymal morphology by forming protrusions and elongating. Collagen pore size and number increase, however, influenced the mechanical properties of MDA-MB-231 cells by reducing the forces exerted by the cells on the ECM networks. These cells showed high traction polarity (a directional force), independent of the type of ECM. Overall, ECM properties and cell traction were correlated. BC cells optimised their mechanical interactions with the ECM to maintain migratory phenotypes. In summary, collagen properties affected the mechanical properties of the BC cells [17] (Figure 2C).

A study used 3D collagen-I networks (with thin or thick fibres) and exposed MCF-7 and MDA-MB-231 cells to them. MDA-MB-231 cells showed a high aspect ratio at greater levels of elastic modulus regardless of fibril thickness status [18]. MCF-7 cells showed increased cluster size for higher modulus in thin fibril networks (thick fibrils reduced cluster size in stiff matrices even with similar elastic moduli). MDA-MB-231 cells also showed increased aggressiveness depending on the thick fibril network elastic modulus. No such relation was detected for thin fibrils, even when the elastic modulus of the thin fibril network was comparable to that of the thick fibril network. The same principle applied to MCF-7. Overall, a stronger invasion capacity was obtained with a higher elastic modulus for thick fibril networks [18]. Finally, at high X-ray irradiation doses (e.g., 320 Gy), the stiffness of the collagen matrix was lower, and it showed liquid-like characteristics. At clinically relevant X-ray doses (e.g., 54 Gy), no significant effects on the viscoelasticity of collagen type 1 were detected [37]. This finding is relevant to clinical and radiological studies.

### 2.2. Basement Membrane

The basement membrane is a nonporous network of the ECM proteins. The main role of the basement membrane is to separate epithelial cells of the tissue from the stromal matrix. It can be seen as a physical barrier, which contains the cancer during early stages. During the invasion phase, the carcinoma cells breach through this membrane [38]. For example, invasive BC can invade the basement membrane [19]. MCF-10A breast epithelial cells were grown on a reconstituted basement membrane to form 3D acini. These acini were harvested to form an interpenetrating network (IPN) of alginate and reconstituted basement membrane. Stiff and soft IPNs represented tumour and healthy tissue, respectively. Soft IPNs had round acini and unaltered basement membranes. Stiff IPNs had invasive acini and breached basement membranes [19]. Protease function was also assessed by adding a metalloprotease inhibitor (GM6001) to the stiff IPN, which reduced cell invasion. Upon breaching the basement membrane and the outward movement of cells, the basement membrane moved inward to the acini. The accumulation and densification of the basement membrane led to the occurrence of holes and actin densification around these sites (Figure 2D). These basement membrane openings showed an increase in focal adhesion kinase (FAK). FAK triggered actomyosin contractility through integrin-induced adhesion. Overall, BC cells used force and proteases to invade the basement membrane. Local contractile forces and forces due to cell volume expansion stretched and breached the basement membrane [19].

### 2.3. Fibroblasts and the ECM

Fibroblasts are core cells of the tumour microenvironment. These cells can contribute to tumour progression by affecting matrix remodelling and deposition [39]. A study formed a tissue-specific 3D microenvironment model comprising the ECM, fibroblasts, and cancer cells. Accordingly, the authors used MCF-7 and T47D (for breast cancer), ACHN and A-498 (for kidney cancer), and PANC-1 and MIA PaCa-2 (for pancreatic cancer) [20]. Von Mises stresses (a measure of stress levels of material) were reported using 3D readouts of stresses along the tissue width (x-axis), tissue length (y-axis), and tissue thickness (z-axis) when the cancer cells were surrounded by fibroblasts and the ECM. In tissues with softer ECMs, such as the pancreas, cancer cells experienced high stress levels compared to kidney and breast tissue with stiffer ECMs. The higher the invasiveness of tumour cells, the greater the stress they experience [20]. Finally, if the cancer cell was in contact with the ECM rather than a fibroblast, it experienced higher stress levels, suggesting intricate dynamics [20]. In another study, 2D cocultures of MCF-7 cells with human lung fibroblasts in the presence of a fibroblast activator, TGFβ, were formed [21]. The coculture of activated fibroblasts with cancer cells formed a ring of cells along the periphery of the islands (COAFs) [21]. MCF-7-GFP cell tracking showed that COAFs spontaneously segregated, suggesting their spatial distribution. The composition of COAFs included E-cadherin and vimentin, which were localised to the inner areas and the periphery of these islands, respectively. This result suggested that epithelial cells were mainly segregated in the inner area, while fibroblasts were segregated in the outer area [21]. Vinculin (a structural component that stabilises FAK) and inner (negative) traction forces were localised where activated fibroblasts were based, with COAF groups showing reduced migratory capacity [21] (Figure 2E). The implication of BC cell entrapment by fibroblasts was the regulation of invasiveness and migration. Overall, islands featured an intricate interplay between protein localisation and cellular mechanics.

## 3. Changes Induced in BC Cells Following Exposure to Forces

### 3.1. Forces Inducing Epithelial-to-Mesenchymal Transition (EMT), Dormancy, and Stemness

**Figure 3:**
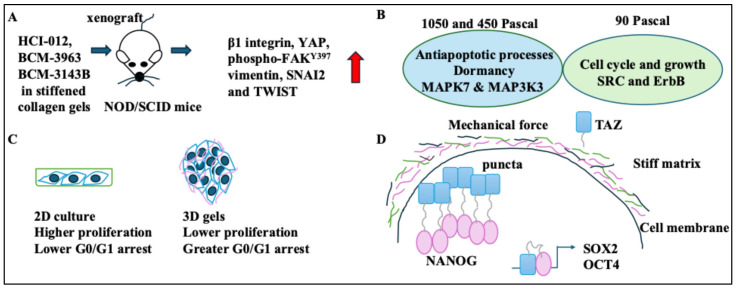
Changes induced in BC cells following exposure to forces. (**A**) 3 HER2+ BC tumours, HCI-012, BCM-3963, and BCM-3143B in stiffened collagen gels, were xenografted to NOD/SCID mice. These tumours grew and increased β1 integrin, YAP, phospho-FAK^Y397^, vimentin, SNAI2, and TWIST levels (red arrow showing increase). (**B**) The 450 and 1050 Pascal groups were associated with MAPK7 and MAP3K3, antiapoptotic processes, and dormancy. The 90 Pascal group was linked to SRC and ErbB, the cell cycle and growth. (**C**) BC cells in 3D gels had lower proliferation rates (and higher G0/G1 arrest rates) than cells isolated from 3D cultures and moved to flasks (which showed higher proliferation and lower G0/G1 arrest rates). (**D**) BC cells in stiff matrices showed high NANOG and TAZ, which activated OCT4 and SOX2.

Stromal stiffness was linked to EMT, defined as migration and mobility programmes in which epithelial cells take a mesenchymal and migratory phenotype. A spatially transformed inferential force map was used to automate atomic force microscopy (with a trained neural network) [22]. 3 HER2+ BC tumours, HCI-012, BCM-3963, and BCM-3143B in stiffened collagen gels, were xenografted to non-obese diabetic/severe combined immunodeficiency (NOD/SCID) mice. Stiff ECMs promoted growth and EMT in these models. These tumours increased β1 integrin, YAP, phospho-FAK^Y397^, vimentin, SNAI2, and TWIST (EMT markers) [22] (Figure 3A). Stiff ECMs contained cross-linked collagen and promoted growth, invasiveness, and EMT in the breast tissue [22,23].

A study shed light on the mechanism of matrix stiffness-induced dormancy in BC cells [24]. Dormancy is the entry of cells into a reversible G0 phase of the cell cycle and has been linked to treatment resistance [40]. The MCF-7 BC cell line was grown in matrices with 90, 450, and 1050 Pascal stiffness levels [24]. In the 450 and 1050 Pascal groups, a decrease in proliferation was observed. This result contrasted with the 90 Pascal group, which showed increased proliferation. The 450 and 1050 Pascal groups were associated with MAPK7 and MAP3K3 and antiapoptotic processes. Despite this, the 90 Pascal group was linked to SRC and ErbB families and the cell cycle and growth. Overall, mechanical forces induced dormancy (increased p38 and decreased ERK) [24] (Figure 3B).

Cells cultured in 3D systems could show stemness and quiescence. Moreover, 4T1, MCF-7, and MDA-MB-231 cell lines were embedded in 3D collagen, fibrinogen, or Matrigel [25]. 45 Pascal forces induced by gels increased colony formation in 4T1 and MCF-7 cells. Accordingly, at 45 Pascals, these cells displayed greater tumourigenesis in Matrigel compared to collagen or fibrinogen gels. In addition, 45 Pascal gel forces also induced integrin β1/3 receptors and AIRE protein levels (an autoimmune regulator expressed by cells in 3D Matrigel) in 4T1, MCF-7, and MDA-MB-231 cells [25]. Contrastingly, 3D Matrigel with 450 Pascal forces drove cell cycle arrest and quiescence via the DDR2/STAT1/P27 pathway [25]. Greater forces by gels (>1000 Pascals) induced apoptosis and damage to tumour cells. As for proliferation, in 4T1, MCF-7, and MDA-MB-231 cell lines cultured in 3D gels, G0/G1 arrest was observed, while isolating these cells from 3D cultures and seeding them in flasks reduced G0/G1 arrest [25]. Overall, ECM-triggered mechanical forces induced quiescence. The greater the gel stiffness, the higher the G0/G1 arrest (Figure 3C). Another study showed that the increased matrix stiffness was associated with BC stem cell enrichment (marked by positive ALDH1 and cytokeratin staining). Based on the cancer stem cell model, cancer stem cells sit at the hierarchy of the ontogeny of the cancer, akin to the hierarchy existing within a normal tissue. These cells can propagate an existing tumour [41]. The abundance of ALDH1^+^CK^+^ tumour cells (BC stem cells) was greater in stiff matrices [26]. Mechanistically, ECM stiffness activated TAZ, and mixing purified mCherry-NANOG and EGFP-TAZ induced phase separation of NANOG. TAZ and NANOG formed a liquid-shaped condensate. In sedimentation assays, MCF-7 and BT-474 cells in stiff matrices expressed NANOG and TAZ in the pellet fraction (phase separation). The TAZ-NANOG phase separation led to OCT4 and SOX2 transcription and stiff matrices induced stemness [26] (Figure 3D).

### 3.2. Changes Induced by Passing Through Narrow Pores

**Figure 4:**
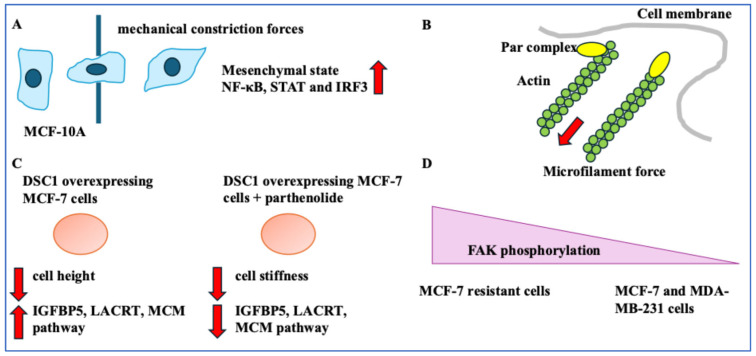
Passing through narrow pores, genes, and diagnostics. (**A**) MCF-10A cells that passed through a capillary showed mesenchymal phenotypes and expressed NF-κB, STAT, and IRF3. (**B**) Par3 and microtubule/microfilament forces were linked (red arrow showing force direction). (**C**) DSC1 overexpressing MCF-7 cells had lower cell height and expressed IGFBP5, LACRT, and the MCM pathway. Treating DSC1-overexpressing MCF-7 cells with parthenolide decreased cell stiffness, IGFBP5, LACRT, and the MCM pathway. Red arrows pointing up or down represent increase and decrease, respectively. (**D**) MCF-7-resistant cells had high FAK phosphorylation compared to MCF-7 and MDA-MB-231 cells.

MCF-10A and MDA-MB-231 interaction on monolayers caused compression forces. In addition to the tension along MCF-10A monolayer cells that formed a tensile belt, interfacial negative curvature (bending of a surface at an interface) induced by finger-like structures caused compression. This compression affected the BC cells surrounded by epithelial cells. This compression led to the extrusion of BC cells and the activation of apoptosis [42]. The mechanical forces experienced by BC cells that have entered the bloodstream are also relevant. Another study formed a microfluidic platform to emulate capillaries using photolithography [27]. MDA-MB-231 and MCF-10A cells were altered to express a nuclear localisation signal (NLS)-GFP tag, and nuclear leakage was represented by cytoplasmic GFP detection. Nuclear rupture occurred when transiting narrow capillaries (5 × 5 μm^2^). MDA-MB-213 and MCF-10A cells remained viable for circa 6 days following transiting a narrow capillary [27]. Constriction forces led to a transient increase in proliferation more profoundly in MCF-10A cells than in BC cells [27]. MCF-10A cells showed affected Lamin A/C organisation. These forces also triggered inflammation pathways such as cGAS/STING, STAT, NF-κB, and IRF3 more profoundly in normal breast tissue cells than in BC cells. MCF-10A cells showed greater transcriptional alterations towards mesenchymal phenotypes than BC cells [27] (Figure 4A). Other authors modelled cell entry through narrow channels in a fluidic domain/phase. They put forth a spring-network model and high-speed imaging method to study the deformability behaviours of MCF-7, SKBR-3, and MDA-MB-231 BC cells. MDA-MB-231 cells showed the greatest deformability [43].

## 4. Genes Linking BC Phenotype and Mechanical Forces, and the Implications for Treatment

Par3 correlated with BC migration. Following exposing MDA-MB-231 cell lines to EGF, TGFβ, and CXCL12, all three molecules increased Par3 tension and microtubule/microfilament forces in MDA-MB-231 cells in wound healing and FRET assays. Par3 and microtubule/microfilament forces were linked to BC aggressiveness. The Par3-siRNA-mediated knockdown in MDA-MB-231 cells reduced cytoskeletal stability and induced cancer invasion [28]. Blocking Par3 and aPKC interaction by inactivating aPKC increased invasiveness in MDA-MB-231 cells in Transwell migration assays. Mechanistically, Par3 tension from microfilament forces triggered inward contractility, which was associated with aPKC loss. Cytoplasmic protein nanoparticle-induced osmotic pressure resulted from Par3 function and microfilament depolymerisation [28] (Figure 4B). Overall, the Par3 protein, the cytoskeleton, and invasion were linked.

Desmocollin-1 (DSC1) is involved in cell-to-cell adhesion [29]. An NF-κB inhibitor (parthenolide) was investigated in the luminal A MCF-7 cell line. Parthenolide decreased DSC1 protein levels in MCF-7 cells. DSC1 overexpression reduced cell height, while parthenolide in these cells decreased cell stiffness. DSC1 overexpression increased insulin growth factor binding receptor 5 (IGFBP5), LACRT, MCM2-7, and CDK2 levels (MCM pathway involved in proliferation). The treatment of DSC1-overexpressing MCF-7 cells with parthenolide decreased these genes [29] (Figure 4C). DSC1 was linked to cell mechanics, proliferation, and migration.

Mechanical forces could impact diagnostics. A study used a noise decorrelation algorithm and image processing comprising quantitative phase imaging to analyse intracellular dry mass (proteins, lipids, carbohydrates, and nucleic acids) [30]. This method detected thermal forces and active ATP utilisation since malignancies expanded these regions to meet their energy demands [30]. The intracellular dry mass fluctuations in MCF-10A, MCF-7, and MDA-MB-231 placed in low ATP concentrations were as follows: MCF-10A non-cancerous cells showed regions of high activity in the cell periphery. MCF-7 cancer cells showed regions of high activity. MDA-MB-231 metastatic cells showed a greater increase in dry mass fluctuations and areas of high metabolic activity. Overall, changes in energy expenditure in metastatic cells could be used to inform diagnostics [30].

Treatment could be impacted by mechanical forces [31]. A study used atomic force microscopy to gain insight into the link between the status of proteins relevant to focal adhesion and drug resistance in BC [31]. This analysis showed actin stress fibres and vinculin were upregulated in MDA-MB-231 and MCF-7 resistant cells (MCF-7/ADR) compared to MCF-7 cells. MCF-7-resistant cells also had higher FAK phosphorylation levels than MCF-7 and MDA-MB-231 cells, suggesting that FAK phosphorylation could be a key factor in disturbing the mechanical forces of BC cells. FAK inhibitors with doxorubicin reduced the IC50 levels and were key to eradicating BC cells. This inhibitor was more effective in MDA-MB-231 and resistant MCF-7 cells [31]. Overall, this study showed the importance of combination treatments with FAK inhibitors (Figure 4D). The post-treatment change in BC and ECM adhesion strength was measured in another study [32]. Accordingly, tamoxifen-treated ER+ BC cells and trastuzumab- and pertuzumab-treated HER2+ cells were included in this analysis. Larger adhesion forces were required to separate treated cells from the ECM. This prevented detachment from the ECM, hence lowering migration. Following chemotherapy treatment, vinculin levels were increased, suggesting tight cell–ECM interactions [32]. Vinculin expression by drug-resistant BCs and BCs following chemotherapy signifies its functional complexity.

Finally, a gradient rotating magnetic field (RMF) can treat TNBC. Gradient RMF affected the F-actin-associated gene, CCDC150 (through the TGF-β1/SMAD3 pathway) in MDA-MB-231, BT-549, and MDA-MB-468 cells and suppressed migration. Gradient RMF and silencing CCDC150 suppressed TNBC metastasis [33]. TNBC can be treated with a magnetic photothermal converter. This method used magneto-mechanical force and near-infrared II hypothermal ablation to target BC cells [34].

## 5. Conclusions

1. Non-invasive BC cells showed viscous membranes but stiffer cells, while metastatic cells showed lower viscous membranes but softer cells. The higher the cell’s malignant potential, the lower the adhesion and the higher the cell deformability [8]. For example, MCF-7 demonstrated greater adhesion and intracellular forces, and stiff phenotypes compared to invasive cells [10,11,12,13]. Overall, this study shed light on the mechanical properties of the BC cells.
2. The ECM also played a role in force dynamics. In the presence of HA, β1-integrin and CD44 were required for traction force generation [15]. This was important since the interplay between transmembrane proteins, GAGs and mechanical forces can inform future studies. Integrin-related mechanical tensions and BC cells during migration were also significant [16]. Collagen pore size and number increase influenced the mechanical properties of MDA-MB-231 cells by reducing the forces exerted by the cells on the ECM [17]. This finding applies to the composition of various hydrogels and scaffoldings used in BC studies. A stronger invasion capacity was obtained for a higher elastic modulus for thick fibril networks [18]. This directly impacted invasive phenotypes and therefore is significant. BC cells also used force and proteases to invade the basement membrane, as shown by others [19,44]. If the cancer cell was in contact with the ECM rather than a fibroblast, it experienced higher stress levels. In addition, TGFβ-activated fibroblasts could form a ring around the BC cells, modulating their movement [20,21]. Both aspects can be considered when designing systems to emulate the BC tumour microenvironment.
3. Changes in BC cells following force exposure were discussed. HER2+ BC xenograft tumours in stiffened collagen gels expressed markers of EMT and β1 integrin [22]. This indicates a direct link between stiffened matrices and increased cell motility. The MCF-7 cells grown in higher Pascal force matrices became dormant. As such, the greater the stiffness of the gel, the greater the G0/G1 arrest and the potential for dormancy [24,25]. These aspects were particularly interesting when considering in vivo changes that induce dormancy following “mechanosensing” [45]. BC cells in stiff matrices showed high NANOG and TAZ levels and stemness [26]. In other words, the niche stiffness could impact cancer stemness in BC cells, and this was orchestrated by a host of key stemness transcriptional regulators. On a side note, changes to BC cells following transit through narrow capillaries were discussed. As a result, nuclear rupture of BC cells occurred. These constriction forces triggered inflammatory pathways and changed proliferation and nuclear stiffness. Cells also became more deformable [27,43].
4. Genes linked BC phenotypes with mechanical forces. Inhibiting the interaction between Par3 and aPKC increased BC cell migration. Inhibiting NF-κB suppressed DSC1 levels and proliferation and affected cell stiffness [28,29]. This suggested gene expression, the cytoskeleton, and important BC cell activities, including migration and proliferation, were linked to the mechanical properties of BC cells. Finally, implications for diagnostics and treatment were assessed. Image processing and intricate algorithms could be diagnostic tools to detect cells with unusually high metabolic needs [30]. Also, vinculin and FAK activity could be BC treatment resistance indicators. The implications of cell–ECM junction tightness and high vinculin were limited migration post-treatment [31,32]. The dual role of vinculin in these two scenarios indicates the complexity of its function in the cell. Mechanical methods can suppress metastasis and alter the disease progression route [33,34]. Overall, understanding the mechanics of BC cells and the ECM will improve treatment.

## Figures and Tables

**Table 1:** The summary of the players and mechanisms encapsulated in this study.

Players	Mechanism	References
Non-invasive and metastatic BC cells	Non-invasive cells had viscous membranes but were stiff, and metastatic cells had less viscous membranes but were soft.	[8,9]
MCF-10A compared to MCF-7 and MDA-MB-231 cells	Highest Young’s modulus (stiffness) in non-malignant cells.	[11]
HA, traction force, β1-integrin in HA gels, and CD44	Blocking CD44 or β1-integrin using antibodies in H2 conditions (collagen I and higher HA gels) reduced traction forces.	[15]
Integrin, adhesion, and EGF	With EGF, cells became more polarised, and it affected integrin-mediated adhesion and cell migration.	[16]
Collagen properties in hydrogels	Collagen pore size and number affected the mechanical properties of MDA-MB-231 cells by reducing the forces these cells exerted on the ECM.	[17]
MDA-MB-231 cells and aggressiveness	The higher elastic modulus for thick fibril networks induced a stronger invasion capacity in MDA-MB-231 cells.	[18]
Basement membrane breach, FAK, forces, and proteases	Basement membrane openings had high FAK activity; BC cells used force and proteases to stretch and invade the basement membrane.	[19]
Softer and stiffer ECMs, BC cells, and invasion	In tissues with softer ECMs, cancer cells experienced high stress compared to stiffer ECMs.	[20]
Coculture of fibroblasts with BC cells (COAFs)	Vinculin and inner negative traction forces were localised to activated fibroblasts, while COAFs had low migratory capacity.	[21]
Stiff ECMs, invasion, and EMT	Stiff ECMs promoted growth, invasiveness, and EMT. These tumours increased β1 integrin, YAP, FAK^Y397^, vimentin, SNAI2, and TWIST.	[22,23]
Matrices with 450 and 1050 Pascal forces or 90 Pascal forces	450 and 1050 Pascal groups showed low proliferation, and 90 Pascal groups showed high proliferation.	[24]
3D Matrigel with 450 Pascal (high stiffness)	The greater the stiffness of the gel, the higher the G0/G1 arrest; 3D Matrigel with 450 Pascal forces drove cell cycle arrest and quiescence.	[25]
Stiff matrices and BC cells	The abundance of ALDH1^+^CK^+^ (stem cells) was greater in stiff matrices.	[26]
Constriction forces, proliferation, and BC cells	Constriction forces increased proliferation and Lamin A/C reorganisation in MCF-10A cells more than in BC cells.	[27]
Par3, the cytoskeleton, and invasion	The Par3-siRNA-mediated knockdown in MDA-MB-231 cells reduced cytoskeletal stability and induced cancer invasion.	[28]
NF-κB inhibitor (parthenolide) and DSC1	Parthenolide decreased DSC1 levels in BC cells. DSC1 expression reduced cell height, and parthenolide decreased stiffness in these cells.	[29]
MDA-MB-231 metastatic cells and metabolism	MDA-MB-231 metastatic cells showed a greater increase in dry mass fluctuations and areas of high metabolic activity.	[30]
MDA-MB-231 cells, MCF-7 resistant cells, and MCF-7 cells	Actin stress fibres and vinculin were high in MDA-MB-231- and MCF-7-resistant cells compared to MCF-7 cells.	[31]
Post-treatment BC cells	In post-treatment BC cells, large adhesion forces were needed to separate cells from the ECM, migration was low, and vinculin was high.	[32]
Gradient RMF and magnetic photothermal converter	Gradient RMF affected the CCDC150 gene in BC cells and suppressed migration. TNBC was treated with a magnetic photothermal converter.	[33,34]

## Abbreviations

Cx43	connexin 43
DSC1	desmocolin-1
ECM	extracellular matrix
EMT	epithelial-to-mesenchymal transition
ER+	oestrogen receptor-positive
FAK	focal adhesion kinase
GAG	glycosaminoglycan
HA	hyaluronic acid
HER2+	human epidermal growth factor receptor-positive
IGFBP5	insulin growth factor binding receptor 5
IPN	Interpenetrating network
nN	Nanonewton
NLS	nuclear localisation signal
NOD/SCID	non-obese diabetic/severe combined immunodeficiency
PR+	progesterone receptor-positive
QPI	quantitative phase imaging
TGT	tension gauge tethering
TN−	triple-negative
